# The Roles of STAT3 and STAT5 in Breast Cancer

**DOI:** 10.3390/cancers17111781

**Published:** 2025-05-26

**Authors:** Alexandra E. Temple, Sarah R. Walker

**Affiliations:** Department of Molecular, Cellular, and Biomedical Sciences, University of New Hampshire, Durham, NH 03824, USA; alexandra.temple@unh.edu

**Keywords:** STAT3, STAT5, breast cancer, TNBC, competition, cofactors

## Abstract

STAT3 and STAT5 are two related transcription factors involved in normal mammary gland development and function. However, inappropriate activation of either STAT3 or STAT5 has been shown to play a role in breast cancer, where STAT3 is highly associated with aggressive tumors and STAT5 is associated with lower-grade and more differentiated tumors. As transcription factors, STAT3 and STAT5 transcriptionally regulate genes involved in proliferation, migration, and chemoresistance. Furthermore, STAT3 and STAT5 transcriptional activity can be modulated by several known cofactors, where these cofactors can influence how STAT3 and STAT5 interact with DNA and with other proteins, ultimately affecting transcriptional function. Interestingly, STAT3 and STAT5 share a subset of overlapping target genes and can compete for DNA binding of shared binding sites. These STATs have also been shown to have opposing effects on overlapping target gene expression, where gene expression is determined by the STAT protein occupying the promoter. This is particularly interesting since STAT5-driven breast tumors are molecularly distinct from STAT3-driven breast tumors. Furthermore, concurrent activation of STAT3 and STAT5 is associated with more favorable tumor types compared to tumors with activated STAT3 alone, suggesting that the relationship between these two STATs is critical. Developing a better understanding about the roles that STAT3 and STAT5 play in breast cancer will be important for successful treatment in the future.

## Simple Summary

Breast cancer affects around 13% of women worldwide, and there is an ongoing effort to understand how different drivers of breast cancer can be exploited for therapeutic targets. Signal transducer and activator of transcription 3 (STAT3) and STAT5 are both highly involved in breast cancer; however, STAT3 activity is often associated with more aggressive subtypes. It has been suggested that STAT5 activation can attenuate STAT3-driven breast tumors, offering an interesting avenue to potentially target STAT3 and use STAT5 as a prognostic marker. This review aims to summarize the recent discoveries regarding the roles that both STAT3 and STAT5 play in breast cancer, specifically focusing on their transcriptional functions, the cofactors involved in regulating their transcriptional activity, and their direct relationship with each other.

## 1. Introduction

### 1.1. Breast Cancer

An estimated 13% of women worldwide are diagnosed with invasive breast cancer, and it is currently the second leading cause of cancer-related deaths worldwide [1]. Over 300,000 new cases of invasive breast cancer were expected in the US in 2024 [2]. Breast cancer incidence has risen over the past two decades and has been attributed to increased body-mass index, reduced fertility rates, and improved diagnostic techniques [1]. Risk factors for breast cancer can include family history, BRCA mutations, obesity, and nonpregnant women with no history of pregnancy [3,4]. Certain lifestyle choices, such as diet and exercise, can help to reduce the likelihood of developing breast cancer. Minimizing alcohol intake can also reduce the risk of developing breast cancer [4]. Breast cancer is generally classified into four distinct molecular subtypes, namely luminal A, luminal B, HER2-positive, and triple-negative breast cancer (TNBC or basal-like), and are characterized by the lack or presence of estrogen receptor (ER), progesterone receptor (PR), and human epidermal growth factor receptor 2 (HER2) [5]. TNBC accounts for about 15–20% of diagnosed breast cancer cases and is classified by the lack of ER, PR, and HER2 expression [6]. TNBC is often aggressive, with poor prognoses, higher grade tumors, and limited treatment options; the standard course of treatment is chemotherapy and immunotherapy. Current research is aimed at finding novel avenues to treat patients with TNBC [7]. Conversely, ER-positive tumors (luminal A) account for the majority of diagnoses and are often less aggressive and demonstrate a better response to treatment [8]. Given that breast cancer affects so many people worldwide, it is important to understand the molecular drivers underlying this disease so that novel biomarkers can be discovered, and treatments can be optimized for successful results.

### 1.2. STAT Family and Function

**Figure 1:**
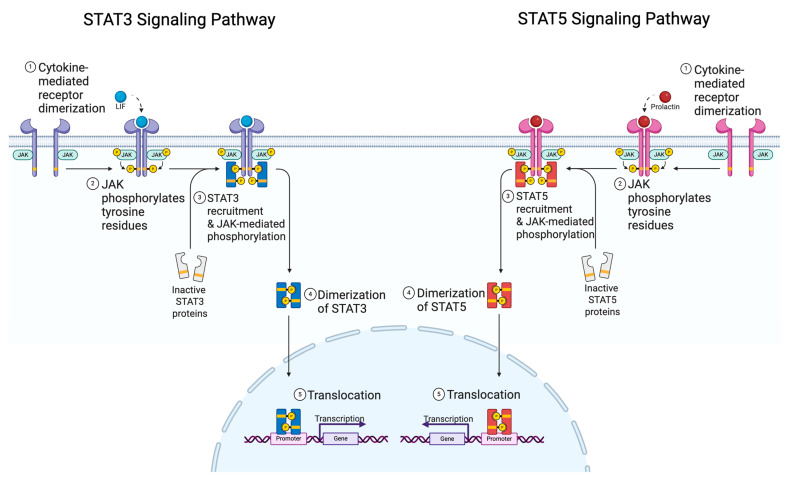
JAK/STAT signaling pathway schematic. Cytokine or growth factor interacts with receptors, resulting in phosphorylation of Janus kinase (JAK). JAK mediates the phosphorylation of STAT monomers, which can subsequently dimerize and enter the nucleus to regulate transcription of target genes.

The signal transducer and activator of transcription (STAT) family comprises seven transcription factors, STAT1, STAT2, STAT3, STAT4, STAT5A, STAT5B, and STAT6. Although STAT5A and STAT5B are two distinct genes, their functions overlap considerably and will generally be referred to as STAT5 for the remainder of this review, unless stated otherwise. These STAT proteins are a family of transcription factors that canonically function as part of the JAK/STAT signaling pathway (Figure 1). Briefly, a cytokine or growth factor binds to its respective receptor, resulting in the phosphorylation of the tyrosine kinase and Janus kinase (JAK). The activated JAK protein then phosphorylates the inactive STAT monomer on a conserved tyrosine residue. These phosphorylated STATs can then homo- or heterodimerize and translocate to the nucleus, where the activated STAT dimer binds to the gamma interferon activation site motif, TTCNNNGAA, to regulate gene expression of select target genes. STAT proteins, especially STAT3 and STAT5, play a role in a variety of biological processes involved in proliferation, differentiation, apoptosis, and immune response [9]. STAT signaling is tightly controlled through regulatory mechanisms to coordinate the action of these transcription factors. The well-known regulatory mechanisms include suppressor of cytokine signaling (SOCS) proteins, protein inhibitors of activated STAT (PIAS), and phospho-tyrosine phosphatases [9], which function as part of a negative feedback mechanism to inhibit STAT activity. Although these STATs play various roles in normal cellular function, dysregulation can contribute to the onset and progression of many cancers, including breast cancer [10]. Specifically, STAT3 and STAT5 have been extensively researched in the context of breast cancer due to their important roles in normal mammary gland development and function. This review will focus on STAT3 and STAT5 in normal breast physiology and breast cancer, and the competing roles they play in normal and malignant development, specifically focusing on their role as transcription factors.

### 1.3. JAK Specificity and Targetability

The JAK family of kinases consists of JAK1, JAK2, JAK3, and TYK2. In normal mammary gland development and breast cancer, JAK2 is the primary activator of both STAT3 and STAT5 [11,12]. Although JAK2 is involved in STAT3 and STAT5 activation, the upstream cytokine and receptor generally offer specificity for which STAT is activated [13]. JAK2 amplification has been found in some cases and is associated with reduced recurrence-free and overall survival in TNBC [14]. Inhibition of JAK kinases has been employed as a therapeutic target for breast cancer treatment [15]. The JAK inhibitor Ruxolitinib has been used in clinical trials for breast cancer (NCT01594216, NCT01562873, and NCT02120417) but has shown subpar results both alone and in combination with other therapeutic agents [16,17,18]. In addition to JAK/STAT signaling, STATs are involved in other signaling pathways. Specifically, STAT3 can be phosphorylated and activated by other noncanonical pathways, such as integrin signaling [19], suggesting that direct targeting of STAT3 could be more efficacious than JAK inhibitors.

## 2. STAT3 and STAT5 in Normal Breast Physiology

### 2.1. STAT3 and STAT5 in Embryogenesis, Puberty, and Menopause

**Figure 2:**
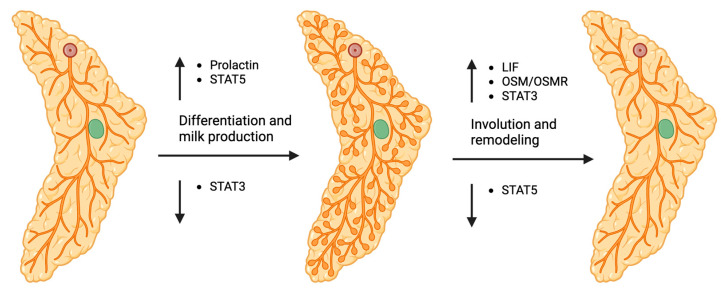
Mammary gland development through pregnancy, lactation, and involution. STAT5 is activated during pregnancy and lactation to drive differentiation and milk production. STAT3 is activated during involution to drive mammary cell death and breast remodeling to a pre-pregnancy state. Up and down arrows indicate increased or decreased expression/activity.

The mammary gland is initially formed as a basic structure during embryonic development, where ductal structures begin to form. During embryogenesis, STAT3 is essential for embryonic growth and development, where STAT3 knockout experiments result in embryonic lethality [20]. The next major stage of mammary gland development occurs during puberty, when estrogen and progesterone hormones from the ovaries signal for ductal elongation, expansion, and invasion into the fat pad [21]. Hormonal changes during the reproductive cycle results in subtle fluctuations of ductal branching. However, complete maturation of the mammary gland does not occur until pregnancy, where progesterone and prolactin drive mammary epithelial cell differentiation, resulting in functional milk-producing glands [21]. STAT5 is highly involved in milk production and differentiation of the mammary gland during pregnancy and lactation (Figure 2), where impaired STAT5 function reduces milk production and lactation. Upon weaning, lactation is halted, and involution begins, where the mammary gland undergoes rapid cell death to return to its pre-pregnancy state. Involution is driven by the deactivation of STAT5 and the activation of STAT3 (Figure 2). The last major change in mammary gland structure and function is during menopause. Major characteristics of menopause include rapid reduction of estrogen production, increased adipose tissue, and regression of the milk-producing lobules [22]. Due to the rapid reduction of estrogen during menopause, many breast cancer studies will separate pre- and post-menopausal women to mimic high and low estrogen levels. STAT3 and STAT5 specifically have been studied extensively during pregnancy, lactation, and involution. STAT5 generally encourages pro-survival and pro-differentiation in breast tissue, whereas STAT3 mediates apoptosis. The distinct roles of STAT3 and STAT5 throughout pregnancy highlight their opposing functions.

### 2.2. Role of STAT5 in Pregnancy and Lactation

When STAT5 was first discovered, it was coined the mammary gland factor due to its role in milk production. However, it was later denoted as STAT5 because of the high similarity between STAT5 and the other STAT family members. STAT5 plays a major role in the mammary gland where STAT5 is activated during pregnancy and lactation, and it is involved in epithelial cell proliferation and differentiation, and milk production [23]. STAT5 is activated by prolactin secretion, and as pregnancy progresses, STAT5 expression and transcriptional activity increase, allowing for the sustained expression of its target genes. Specifically, STAT5 is essential for the production of milk proteins such as whey acidic protein and beta-casein through direct transcriptional regulation of the corresponding genes [10]. Inadequate STAT5A activation leads to reduced milk gene expression and milk production; however, in the absence of STAT5A, STAT5B can be upregulated [24]. Furthermore, studies have demonstrated the importance of STAT5 in alveolar cell differentiation and survival, where failure to activate STAT5 leads to reduced alveologenesis [10,25], which results in diminished milk production. Upon weaning and subsequent milk stasis, STAT5 activity is quickly halted, and STAT3 expression, phosphorylation, and transcriptional activity is upregulated to promote mammary cell death.

### 2.3. Role of STAT3 in Involution and Post-Lactational Remodeling

STAT3 is a critical regulator of cell death of the mammary epithelium during involution. Involution refers to the mammary cell death following weaning and milk stasis and comprises a reversible phase and an irreversible phase. Milk stasis is considered to be a major trigger of involution [26], and leukemia inhibitory factor (LIF)-induced activation of STAT3 [27,28] mediates involution through lysosomal-mediated programmed cell death [10,29]. STAT3 is initially activated through LIF stimulation and maintains its active state through subsequent upregulation of the oncostatin M receptor (OSMR), resulting in oncostatin M (OSM)-induced activation of STAT3 [30], where OSM is the primary cytokine driving STAT3 activation during the irreversible phase [29]. Interestingly, STAT5 phosphorylation and milk gene expression are inhibited by OSM signaling but not LIF [30], suggesting that STAT5 dephosphorylation is promoted through activation of the OSM/OSMR-STAT3 signaling pathway during involution. This could also suggest that there is a brief period before upregulation of OSMR and inhibition of STAT5 activity where both STAT5 and LIF-induced STAT3 are active. Although this warrants further study, this could provide helpful information to understand the interplay between STAT3 and STAT5 in breast cancer. STAT3 is also involved in the upregulation of acute-phase response gene expression and modulation of the immune response in mammary epithelium at the onset of involution [31,32]. Conditional knockout of STAT3 in the mouse mammary gland resulted in delayed involution and reduced cell death [33], demonstrating its importance in involution and post-lactational remodeling of the mammary gland, where STAT3 serves as an apoptotic mediator at the onset of involution. Interestingly, STAT5 protein levels remained higher during involution with conditional knockout of STAT3 compared to wildtype, providing further evidence for a competitive role between STAT3 and STAT5 that can be exploited for future therapies.

## 3. STAT5 in Breast Cancer

### 3.1. Role of STAT5 in Breast Cancer

While STAT5 is crucial for terminal mammary gland differentiation during pregnancy and lactation, STAT5 signaling and transcriptional activity can be hijacked to promote disease initiation and progression in breast cancer. STAT5 can be activated by several cytokines or growth factors in breast cancer, including prolactin (PRL), growth hormone, epidermal growth factor (EGF), and insulin-like growth factor (IGF) [24]. Activation of STAT5 through the JAK/STAT signaling pathway results in tyrosine phosphorylation of either Y694 (STAT5A) or Y699 (STAT5B), which allows for dimerization and subsequent translocation to the nucleus to regulate gene transcription. STAT5 is a pro-survival factor in normal mammary gland development, and function and inappropriate activation of STAT5 is often involved in the initiation of breast cancer tumorigenesis. However, STAT5 also promotes mammary epithelial differentiation, and its activity is associated with improved patient outcomes in ER-positive tumors [23]. Additionally, breast cancers with active STAT5 are generally more differentiated and less likely to metastasize [34,35]. STAT5 activation is often reduced in HER2-positive and TNBC cells [36]. STAT5 activation also reduces metastatic potential and hinders migration and invasion of breast cancer cells in vitro and in vivo [37]. Furthermore, STAT5 activity is often reduced or absent during breast cancer progression and metastasis [34,38], suggesting a need for additional oncogenic pathways to promote tumorigenesis. Importantly, prolactin treatment has been shown to limit tumorigenic properties of aggressive subtypes like TNBC [39]. Expression of prolactin receptor in TNBC is also associated with higher overall survival [40]. This raises the possibility that activating STAT5 in TNBC could be a useful treatment strategy to explore in future studies. Therefore, it is important to understand the role that STAT5 plays in breast cancer and how it can be utilized as a potential marker of disease progression and as a therapeutic target for the treatment of breast cancer.

### 3.2. STAT5 Transcriptional Regulation of Genes

In breast cancer, STAT5 regulates transcription of specific target genes, where STAT5 has both oncogenic and inhibitory functions. STAT5 promotes tumorigenesis through upregulation of genes that are involved in proliferation, migration, and invasion (Table 1). It has been demonstrated that STAT5A is required for expression of NADPH oxidase 5 long form (*NOX5-L*) in HER2-positive SK-BR-3 breast cancer cells where *NOX5-L* inhibition reduced proliferation, migration, and invasion [41]. STAT5 has also been shown to promote therapeutic resistance in some breast cancer cells. For example, STAT5A promotes doxorubicin resistance through transcriptional upregulation of ATP binding cassette subfamily B member 1 (*ABCB1*) in breast cancer [42]. Additionally, STAT5 has been found to promote metastatic potential through transcriptional upregulation of *FYN*. Specifically, a feedback loop was proposed in MDA-MB-231 TNBC xenograft tumors, suggesting that STAT5 promotes *FYN* gene expression. FYN then promotes STAT5 phosphorylation and subsequent downstream NOTCH2 pathway activation [43]. This feedback loop between STAT5 and FYN was shown to increase metastatic potential of the tumors [43]. This research emphasizes the oncogenic role of STAT5 in breast cancer. However, given the well-differentiated, less aggressive tumor types that STAT5 is often found in, there is evidence that STAT5 can act as a suppressor of oncogenic activity, at least in part, to reduce tumorigenic potential. Specifically, STAT5 can suppress migratory and invasive potential, reduce metastasis, and improve response to treatment [23,44]. STAT5A has been found to be negatively correlated with interferon-stimulated exonuclease gene 20 (ISG20) expression in breast cancer, where ISG20 is upregulated in metastases and is associated with aggressive and invasive tumor behavior. STAT5A expression can reduce ISG20 protein levels through modulation of members of the miR-17-92 microRNA cluster, inhibiting invasive potential [45]. Specifically, silencing STAT5A resulted in a reduction of this microRNA cluster, followed by an increase in ISG20 protein expression. STAT5 has been shown to transcriptionally regulate the miR-17-92 cluster in normal mammary tissue [46], suggesting that STAT5 can act as a tumor suppressor by promoting miR-17-92 expression, which results in reduced ISG20 expression. Prolactin-induced STAT5 can also repress expression of B-cell lymphoma 6 (*BCL6*) in breast cancer cells through direct binding to the gene [47]. Furthermore, nuclear localization of STAT5A was negatively correlated with *BCL6* expression in breast cancer tissue samples, where *BCL6* expression is associated with increased proliferation and cell cycle progression and reduced differentiation [48]. This suggests that STAT5 can inhibit breast cancer progression through modulation of *BCL6*. A previous study demonstrated that loss of nuclear tyrosine-phosphorylated STAT5 could predict the failure of tumors to respond to anti-estrogen therapies [38], suggesting that STAT5 can regulate expression of genes that are important for therapeutic response. There are also studies that describe prolactin and its receptor as important factors in reducing tumorigenesis [49,50], further implicating STAT5 in the suppression of tumorigenic properties. However, there are not many studies that look explicitly at the STAT5-regulated genes that are important for the more favorable characteristics often seen in tumors expressing STAT5. The potential for STAT5 as a biomarker in breast cancer has been previously suggested [51,52], but further research needs to be conducted to fully elucidate how STAT5 can act as both a tumor promoter and tumor suppressor in various subtypes of breast cancer. Higher STAT5 expression has been associated with increased recurrence-free survival, whereas high STAT3 expression has been associated with a worse recurrence-free survival [52]. Although STAT5A and STAT5B are often grouped together as STAT5, it is important to note that some differences between the two have been shown in normal mammary gland and breast cancer [53,54,55,56]. STAT5B has been implicated in migration [56], whereas loss of STAT5A was associated with disease progression [54], highlighting the importance in future studies to distinguish between the two STAT5 proteins to enrich clinical efficacy of STAT5 as a biomarker for breast cancer. Given the contradictory role of STAT5 in breast cancer, further research is needed to completely understand the role of STAT5 as a tumor promoter and a tumor suppressor, particularly if using STAT5 as a biomarker or treatment strategy.

### 3.3. STAT5 Protein Interactions in Breast Cancer

**Figure 3:**
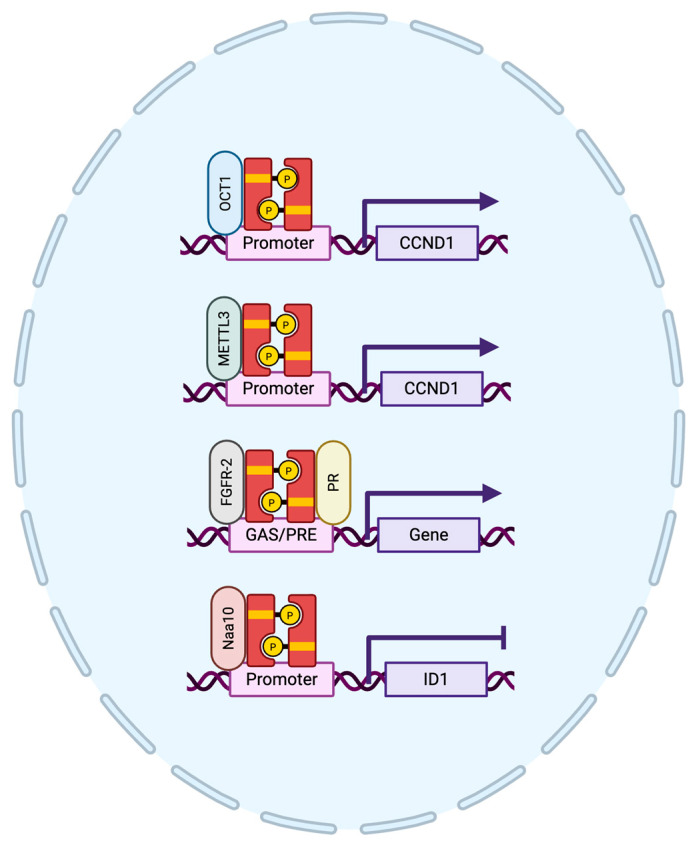
Simplified schematic of STAT5 cofactors involved in STAT5 transcriptional regulation of target genes. Upon cytokine or growth factor stimulation, STAT5 is activated through tyrosine phosphorylation and enters the nucleus to regulate transcription of target genes. STAT5 interacts with different cofactors to modulate gene expression or target genes.

STAT5 has been shown to interact with many proteins, and these functional interactions that play a variety of roles within the cell have previously been reviewed [57]. Here, we will focus on the recently identified protein interactions that directly affect STAT5 transcriptional activity and DNA binding in breast cancer (Table 1). Some STAT5-interacting proteins have been shown to enhance STAT5-mediated gene expression, whereas others have been found to antagonize STAT5 transcriptional activity (Figure 3). Octamer transcription factor 1 (OCT1) is a transcription factor that has previously been shown to form a complex with STAT5. Specifically, OCT1 interacts with the transactivation domain of STAT5A to promote prolactin-induced cyclin D1 expression [58,59], which encourages cell cycle progression in breast cancer. Furthermore, METTL3 has been found to interact with the transactivation domain of EGF-induced STAT5B in the nucleus of breast cancer cells. Specifically, METTL3 association with the *CCND1* (cyclin D1) promoter region was reduced with STAT5B knockdown, but not STAT5A, which impaired cell cycle progression in hormone receptor-positive MCF7 breast cancer cells [60]. This suggests that both STAT5A and STAT5B promote cyclin D1 expression in breast cancer through interacting with different cofactors. Recently, a study demonstrated that fibroblast growth factor receptor 2 (FGFR-2) interacts with progesterone receptor (PR) and STAT5 in hormone receptor-positive T47D breast cancer cells and in breast cancer patient samples. FGF-2 and medroxyprogesterone acetate (MPA) stimulation prompted the colocalization of FGFR-2, PR, and STAT5 in the nuclei of breast cancer cells. Luciferase constructs containing either a gamma interferon activation site (GAS) sequence for STAT5 activity or a progesterone response element (PRE) for PR activity also demonstrated an increase in transcriptional activity for both STAT5 and PR when stimulated with FGF-2 or MPA. This could be abolished via the addition of either the anti-progestin, Lonaprisan, or an FGFR inhibitor, suggesting that the colocalization of FGFR-2, PR, and STAT5 is indeed needed for proper STAT5 transcriptional activity [61]. There has also been evidence of protein interactions that can have an antagonistic effect on STAT5 transcriptional activity in breast cancer. For example, N-a-acetyltransferase 10 protein (Naa10p) has been shown to interact with STAT5A in hormone receptor-positive MCF-7 cells and inhibits STAT5-mediated gene expression of the target gene, *ID1*. Loss of Naa10p resulted in increased *ID1* expression and breast cancer cell migration, whereas knockdown of STAT5 demonstrated reduced *ID1* gene expression and inhibited cell migration [62]. Another protein, high mobility group nucleosome binding domain 2 (HMGN2), can modulate STAT5 transcriptional activity by increasing chromatin accessibility for STAT5 DNA binding to the chromatin [63]; however, a direct protein interaction between STAT5 and HMGN2 has not yet been established. Specifically in breast cancer, HMGN2 can facilitate the loss of linker histone H1 at the *CISH* promoter region, allowing for STAT5 DNA binding and subsequent transcriptional activity [64]. Therefore, understanding how STAT5 cofactors contribute to either the tumor-promoting or tumor-suppressing functions exhibited by STAT5 activity through modulation of target gene expression will be important for future studies regarding the role of STAT5 in breast cancer.

**Table 1:** Summary table of STAT5 target genes and cofactors in breast cancer.

Name	Target Gene or Cofactor	Clinical Relevance	STAT5 Function
*NOX5-L* [41]	Target gene	Proliferation, migration, invasion	Upregulates
*ABCB1* [42]	Target gene	Chemoresistance	Upregulates
*FYN* [43]	Target gene	Metastatic potential	Upregulates
ISG20/miR-17-92 [45,46]	Target gene	Metastatic potential	Downregulates
*BCL6* [47,48]	Target gene	Proliferation, cell cycle progression	Downregulates
OCT1 [58,59]	Cofactor	Cell cycle progression	Promotes
METTL3 [60]	Cofactor	Cell cycle progression	Promotes
FGFR-2 [61]	Cofactor	Proliferation	Promotes
PR [61]	Cofactor	Proliferation	Promotes
Naa10p [62]	Cofactor	Inhibits migration, STAT5-mediated ID1 expression	Inhibitory
HMGN2 [63,64]	Not established	Facilitates chromatin accessibility	Promotes

## 4. STAT3 in Breast Cancer

### 4.1. Role of STAT3 in Breast Cancer

Dysregulation of STAT3 signaling can lead to a variety of diseases, including breast cancer. STAT3 has been implicated in breast cancer tumorigenesis and has been proposed as a potential therapeutic target, where STAT3 is inappropriately activated in approximately 70% of breast cancers [65]. STAT3 can be activated in breast cancer by a number of cytokines and growth factors, including interleukin-6 (IL-6), OSM, IGF, FGF, and EGF [12]. The role of LIF and LIFR in the normal mammary gland has been well-characterized; however, in breast cancer, there are conflicting results. Some studies suggest that higher expression of LIFR is associated with reduced metastasis and improved patient survival [66]. On the other hand, high LIFR expression has been correlated with tumor progression, and inhibition of LIFR improves histone deacetylase (HDAC) inhibitor efficacy [67], suggesting that further research is needed to elucidate the role of LIF and LIFR in breast cancer. STAT3 is often constitutively activated through increased cytokine or growth factor stimulation or decreased STAT3 inhibition by negative feedback inhibitors such as SOCS or PIAS. STAT3 polymorphisms have been reported in breast cancer but were either not significant in tumorigenesis or were controversial [68]. It has been demonstrated that STAT3 plays critical roles in migration, proliferation, epithelial-to-mesenchymal transition (EMT), and chemoresistance, making the STAT3 signaling pathway a desirable target for breast cancer [12]. STAT3 activity is highly associated with TNBC, which is known to have poor patient outcomes due to its lack of effective treatment options and aggressive nature [69]. The role of STAT3 in HER2-positive breast cancers is controversial. Some studies suggest that STAT3 can promote metastatic potential and cancer stem cell properties in HER2-overexpressing breast cancer cells [70,71]. On the contrary, lower phospho-STAT3 has been correlated with HER2-expressing tumors [72], suggesting that further analysis is needed to elucidate the role that STAT3 plays in HER2-positive breast tumors. Direct targeting of STAT3 in vitro has been shown to be efficacious. For example, STAT3 inhibition in MDA-MB-231 TNBC breast cancer cells sensitized cells to doxorubicin treatment, resulting in increased cell death [73]. Additionally, hormone receptor-positive MCF-7 breast cancer cells overexpressing HER2 had higher rates of apoptosis with a combination treatment of Herceptin and the STAT3 inhibitor Stattic, compared to either treatment alone [70]. Tamoxifen-resistant MCF-7 breast cancer cells exposed to Stattic had increased cell death and reduced proliferation [74]. Importantly, another STAT3 inhibitor, TTI-101, has also been studied in a clinical trial addressing many cancer types, including breast cancer (NCT03195699), suggesting its potential as a therapeutic target [75]. Although the complete role of STAT3 in breast cancer has not yet been fully elucidated, these studies provide evidence that STAT3 can be targeted to reduce proliferation and promote breast cancer cell death.

### 4.2. STAT3 Transcriptional Regulation of Genes

In breast cancer, STAT3 functions as a transcription factor that regulates gene expression of several direct target genes involved in proliferation, migration, EMT, and chemoresistance (Table 2). Recently, STAT3 has been found to promote programmed death ligand 1 (*PD-L1*) expression through direct DNA binding and transcriptional activity [76,77]. *PD-L1* silencing resulted in reduced tumor growth, demonstrating the role of STAT3 in promoting proliferation and growth through modulation of *PD-L1* gene expression. The involvement of STAT3 in EMT has also been studied. IL-6-induced STAT3 activation has been shown to promote invasiveness and metastasis through *Twist* expression, where increased Twist expression can promote metastasis, EMT, and cell survival [78]. Additional studies have shown that STAT3 can bind to the promoter region of estrogen-related receptor α (*ERRα*), resulting in increased expression of *ERRα* [79]. Simultaneous STAT3 overexpression and *ERRα* knockdown increased E-cadherin and decreased mesenchymal markers, indicating that *ERRα* is an important STAT3 target for the promotion of EMT in breast cancer. STAT3 also has been shown to bind to the gene encoding *TNFRSF1A* which can upregulate NF-kB signaling in TNBC [80], ultimately promoting breast cancer cell survival and proliferation. Interestingly, STAT3 can directly regulate transcription of high mobility group nucleosome binding domain 5 (*HMGN5*), a protein involved in chromatin remodeling and accessibility [81]. HMGN5 is involved in tumorigenesis and is considered to be an unfavorable marker in breast cancer. In addition to STAT3 upregulation of HMGN5 expression, HMGN5 was also required for STAT3-mediated chromatin accessibility. Many studies have also implicated STAT3 in chemoresistance and reduced therapeutic response. For example, leptin-induced STAT3 transcriptionally regulates expression of carnitine palmitoyltransferase-1B (*CPT1B*) mRNA to promote CPT1B protein expression, which enhances fatty acid β-oxidation. This results in the promotion of chemoresistance and cancer cell stemness in breast cancer cells. Additionally, knockdown of STAT3 could downregulate fatty acid oxidation and reduce self-renewal potential [82]. While these are only some examples, STAT3 transcriptional activity is highly involved in breast cancer tumorigenesis, and identifying important STAT3 target genes will aid in better understanding the role STAT3 plays in breast cancer.

### 4.3. STAT3 Protein Interactions in Breast Cancer

**Figure 4:**
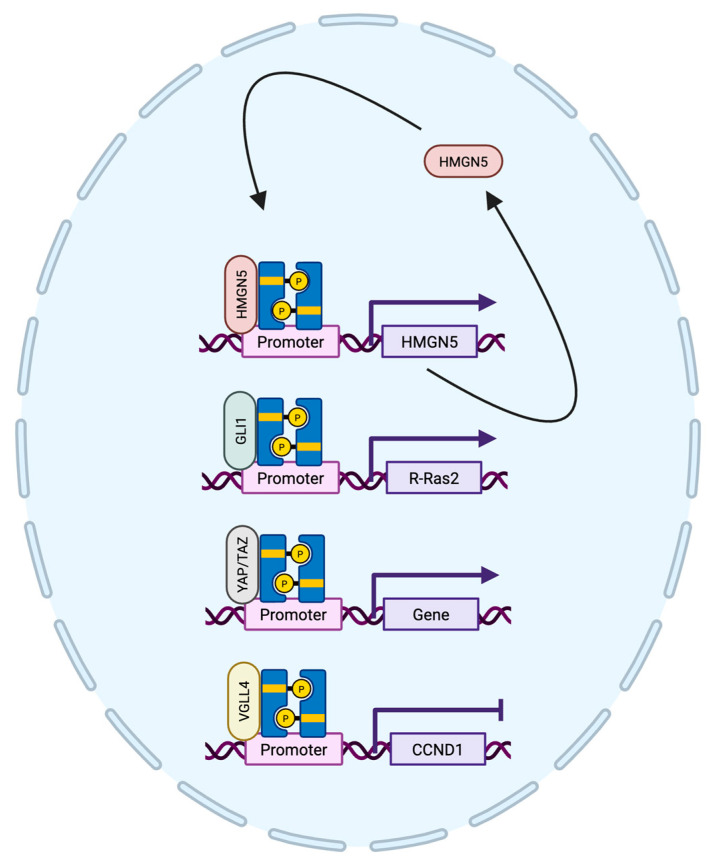
Simplified schematic of STAT3 cofactors involved in STAT3 transcriptional regulation of target genes. Upon cytokine or growth factor stimulation, STAT3 is activated through tyrosine phosphorylation and enters the nucleus to regulate transcription of target genes. STAT3 interacts with different cofactors to enhance or repress expression of target genes.

STAT3 has been shown to interact with many different proteins in breast cancer to drive tumorigenesis. Although there are a variety of protein interactions that have been reviewed and are associated with the many functions of STAT3 [83], the following will focus on the recent discoveries and developments about the interacting proteins involved in STAT3 DNA binding and transcriptional activity in breast cancer (Figure 4). The STAT3-interacting proteins that have been reported can either enhance or inhibit STAT3. For example, granulin was discovered to be a novel STAT3-interacting protein that enhances STAT3 activity in breast cancer cells [84]. It was shown that granulin was needed for maximal STAT3 tyrosine phosphorylation and subsequent activation through cytokine stimulation. Furthermore, knockdown of granulin resulted in reduced expression of STAT3 target genes, including *BCL6*. STAT3 has also been shown to interact with glioma-associated oncogene homolog 1 (GLI1), a transcription factor involved in the Hedgehog signaling pathway. Three genes, *R-Ras2*, *Cep70*, and *UPF3A*, were found to be upregulated by the STAT3/GLI1 complex, where expression of these genes is associated with a worse metastasis-free patient survival [71]. As mentioned previously, STAT3 promotes expression of HMGN5. Beyond that, STAT3 and HMGN5 also colocalize in MDA-MB-231 TNBC tumor spheres [81]. Overall, HMGN5 was found to be highly expressed in breast cancer tissue compared to normal tissue and was associated with a worse patient prognosis and outcome. Since MDA-MB-231 xenografts with knockdown of HMGN5 exhibited reduced tumor growth [81], this suggests that the interaction between STAT3 and HMGN5 could be used as a future target for treatment. The downstream Hippo pathway effector yes-associated protein 1 (YAP1) and its paralog, TAZ, have been identified as STAT3 interacting proteins in breast cancer. It was demonstrated that YAP and TAZ act as STAT3 coactivators and could be recruited to STAT3 binding sites in TNBC cell lines [85]. On the contrary, vestigial-like family member 4 (VGLL4), previously known for its role in suppressing TEA domain transcription factor transcriptional activity, has been shown to interact with STAT3 and suppress STAT3 activity in TNBC [86]. Loss or reduction of VGLL4 was seen in breast cancer tissue compared to that of normal tissue. The Hippo signaling pathway is usually modulated by upstream events such as response to cell density, cell polarity, and other mechanical cues [87]. This could suggest a potential role for STAT3 in the Hippo signaling pathway in breast cancer, specifically modulation of STAT3 activity in response to mechanical stimuli rather than chemical stimuli. Lastly, a relationship between STAT3 and PR has been identified, where STAT3 was suggested to be a coregulator of PR activity [88]. Since STAT5 and PR have been found to interact, it would be interesting to see if PR can modulate STAT3 activity or if PR is involved in the opposing roles often seen by STAT3 and STAT5. STAT3 has many cofactors that are required for optimal STAT3 transcriptional activity and could be exploited for use in breast cancer therapies.

**Table 2:** Summary table of STAT3 target genes and cofactors in breast cancer.

Name	Target Gene or Cofactor	Clinical Relevance	STAT3 Function
*PD-L1* [76,77,89]	Target gene	Tumor growth	Upregulates
*Twist* [78,90]	Target gene	Invasion, metastasis	Upregulates
*ERRα* [79]	Target gene	EMT, metastasis	Upregulates
*TNFRSF1A* [80]	Target gene	Survival, proliferation	Upregulates
HMGN5 [81]	Target gene/cofactor	Facilitates chromatin accessibility	Upregulates/promotes
*CPT1B* [82]	Target gene	Chemoresistance	Upregulates
*BCL6* [47]	Target gene	Proliferation, cell cycle progression	Upregulates
GRN [84]	Cofactor	Maximal STAT3 activity	Promotes
GLI1 [71]	Cofactor	Worse metastasis-free survival	Promotes
YAP1/TAZ [85]	Cofactor	STAT3 coactivators in TNBC	Promotes
VGLL4 [86]	Cofactor	Suppress STAT3 activity	Inhibitory

## 5. STAT3 and STAT5 in Breast Cancer

### 5.1. Connection to Clinical Outcome

STAT3 and STAT5 are both activated downstream of the JAK/STAT signaling pathway in response to cytokine or growth factor stimulation. Inappropriate activation of these STATs can occur for a variety of reasons but mainly include either sustained upstream cytokine signaling or downregulation or inhibition of STAT-negative regulators such as SOCS or PIAS. Both STAT3 and STAT5 have been found to be inappropriately activated in breast cancer. STAT3 is activated in about 70% of breast cancers but is most often associated with TNBC [69]. More specifically, constitutive STAT3 activation alone is found in about 40% of cases, and constitutive activation of both STAT3 and STAT5 is found in about 30% of breast cancer cases. On the other hand, STAT5 activation alone is only seen in about 7% of breast cancer cases, suggesting that alternative pathways may be needed to drive tumorigenesis [35]. It has been previously demonstrated through tissue microarrays stained for either tyrosine-phosphorylated STAT3 or tyrosine-phosphorylated STAT5 that activation of these STATs was mainly a property of the tumor cells, and not the surrounding tissue, and that STAT staining was largely seen in the nuclei of tumor cells, most likely indicating canonical STAT function [35]. Furthermore, breast cancer cases that had concurrent activation of STAT3 and STAT5 were more likely to be well differentiated and associated with a more favorable prognosis and tumor type [35]. This suggests that the activation of STAT5 could potentially modulate STAT3-driven tumorigenesis. When MDA-MB-468 TNBC breast cancer cells, which have constitutively active STAT3, were forced to express constitutively active STAT5, the genotypic profile of these cells matched the genotypic profile of tumors expressing both STAT3 and STAT5 [35,65]. These data suggest that it is important to study these STATs together rather than in isolation since STAT5 can seemingly affect the phenotype of tumors with activated STAT3. Given the distinct roles of STAT3 and STAT5 in normal mammary gland development and function, it would be interesting to further dive into the relationship between these two transcription factors and how STAT5 may modulate STAT3 pathogenesis in breast cancer.

### 5.2. STAT3 and STAT5 Can Compete for DNA-Binding in Breast Cancer

**Figure 5:**
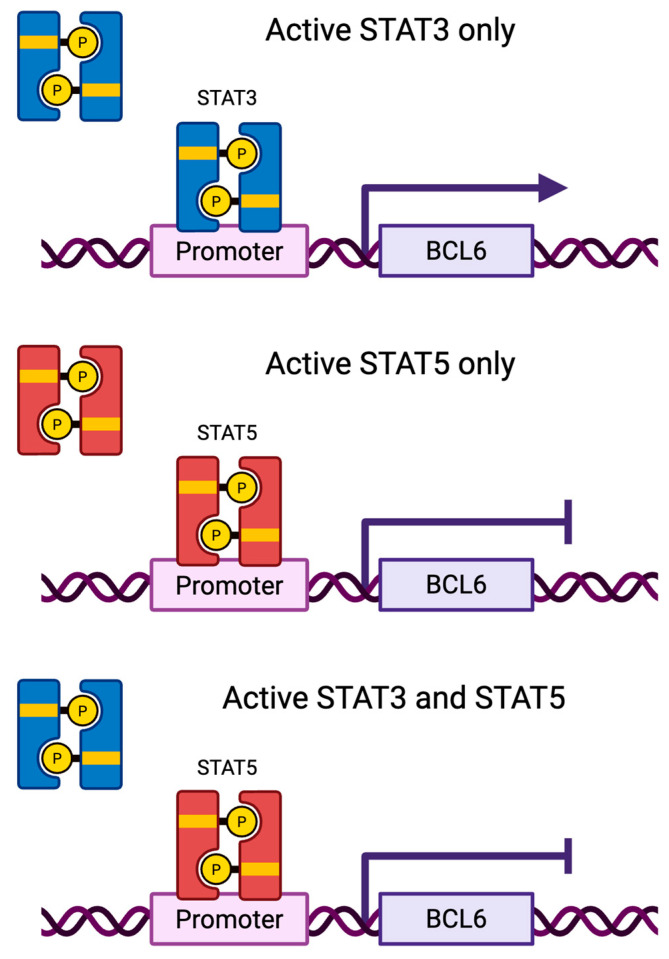
STAT3 and STAT5 compete for transcriptional control of the *BCL6* gene. When both STAT3 (blue) and STAT5 (red) are activated, STAT5 preferentially binds to the promoter and represses *BCL6* gene expression. With the absence of STAT5, activated STAT3 binds to the promoter and enhances *BCL6* gene expression.

STAT3 and STAT5 primarily recognize and bind to the binding motif TTCNNNGAA. Although these two STATs interact with the same binding motif, they both have distinct target genes that they transcriptionally regulate. However, STAT3 and STAT5 can bind to a subset of overlapping genes, where in some cases, it has been demonstrated that STAT3 and STAT5 can compete for DNA-binding and transcriptional control of certain target genes in breast cancer. Specifically, STAT3 and STAT5 have been shown to both bind to the same binding region, Region B, in the *BCL6* promoter region, resulting in transcriptional regulation of the *BCL6* gene (Figure 5) [47]. Furthermore, STAT3 and STAT5 oppositely regulate *BCL6* gene expression, where STAT3 enhances it and STAT5 represses it (Figure 5) [47,48]. Of interest, STAT5 was found to outcompete STAT3 in both transiently and constitutively active forms [47]. Additionally, prolactin-stimulated STAT5 was able to bind to Region B in MDA-MB-468 TNBC cells, which exhibit constitutive STAT3 activity. It was also demonstrated that STAT3 could recruit RNA pol II to Region B, whereas RNA pol II binding to Region B was reduced with prolactin-stimulated STAT5 [47]. C/EBPβ and FOXA1 were analyzed to see whether they could modulate STAT binding to Region B, but neither protein could influence STAT binding. This emphasizes the need for further research to uncover the cofactors that are directly involved in STAT activity and DNA binding. Mechanistically, it is still unknown exactly how STAT5 can outcompete STAT3 for binding to Region B. However, it is possible that the more favorable phenotypic features seen in tumors with concurrent STAT3 and STAT5 activation could be attributed to the competition for overlapping binding sites, like *BCL6*. Information regarding the crosstalk between STAT3 and STAT5, specifically in breast cancer, is limited, and further research on this relationship could help with developing new treatment strategies targeting these STATs, particularly in TNBC, where STAT3 is often constitutively active and STAT5 is often inactive.

### 5.3. STAT3 and STAT5 Relationship in Other Cell Types

Reports of STAT3 and STAT5 with opposing functions and competition have been demonstrated in other cell types. STAT3 and STAT5 have been shown to bind to the IL-17 gene locus and reciprocally regulate interleukin-17 (*IL-17*) expression and Th17 differentiation [91]. Importantly, STAT3 stimulated *IL-17* expression and Th17 proliferation, whereas STAT5 had a repressive effect. Furthermore, protein arginine methyltransferase 1 was required for recruitment of STAT3 to *IL-17*, along with the removal of STAT5 through the addition of the histone modification, di-methylated arginine 3 on histone 4, which was needed for IL6-mediated STAT3 binding and displacement of STAT5 [92]. This potentially suggests that epigenetic marks could help explain global competition between STAT3 and STAT5. However, epigenetic modifications need to be further studied to fully understand their impact on STAT3 and STAT5 transcriptional activity and competition, particularly in the context of competition for *BCL6* in breast cancer. It has also been reported that STAT3 and STAT5 have distinct roles in determining myeloid progenitor cell fate. Granulocyte colony-stimulating factor activates STAT3 to promote neutrophil differentiation, and granulocyte–macrophage colony-stimulating factor activates STAT5 to promote monocyte/macrophage differentiation. Furthermore, STAT5 can inhibit STAT3 phosphorylation and subsequent neutrophil maturation through modulation of *SOCS3* [93]. These studies highlight the opposing nature seen between STAT3 and STAT5 across many cell types.

## 6. Conclusions

Since breast cancer continues to affect so many women worldwide, there is a growing need for novel therapeutic approaches for effective treatment. Given that STAT3 and STAT5 play important roles during normal breast development and pregnancy and are drivers of tumorigenesis, they could offer novel targets for treatment. In the normal mammary gland, STAT3 has a pro-apoptotic function which is required for the mass cell death during involution. However, in breast cancer, STAT3 is a known driver of chemoresistance, EMT, and metastasis, where it transcriptionally regulates genes involved in these processes. On the other hand, STAT5 acts as a pro-survival and differentiation factor during pregnancy and lactation to induce mammary gland growth and milk production. In breast cancer, STAT5 can promote tumor initiation but is often not found in metastases, suggesting the need for additional oncogenic factors. Furthermore, given that STAT5 activation in breast cancer is often associated with well-differentiated and lower-grade tumors, STAT5 activation status could be used as a potential predictor for therapeutic response and patient outcome. Both STAT3 and STAT5 interact with many different proteins to promote or repress transcription of target genes. Developing a better understanding as to how these protein interactions modulate STAT3 and STAT5 transcriptional activity could serve as an avenue for drug development to attenuate STAT3 or STAT5 function. Further research needs to be conducted to better understand how these two related transcription factors can be used in the ongoing efforts to identify novel targets for effective treatments for breast cancer.

## 7. Future Directions

As transcription factors, STAT3 and STAT5 share a distinct set of overlapping target genes. Since tumors containing both constitutively active STAT3 and STAT5 have distinct molecular and phenotypic profiles compared to tumors with constitutively active STAT3 alone, it is possible that the group of overlapping target genes play a considerable role in driving breast cancer severity. It is reasonable to think that for breast tumors containing constitutive STAT3 activity but lacking STAT5 activity, STAT5 could be activated through therapeutic intervention. By activating STAT5 in STAT3-driven tumors, STAT5 could modulate STAT3 activity and promote differentiation, chemosensitivity, and apoptosis. Although STAT5 has been demonstrated to modulate STAT3-transcriptional activity in vitro, further research needs to be conducted in more relevant models, such as 3D scaffolds or STAT5 transgenic mouse models, especially considering that STAT3 may be involved in the Hippo signaling pathway. Additionally, cell line selection should be considered since different cell lines are derived from different tumor subtypes and could have an impact on STAT activity. Exploring combinatorial approaches of STAT3 inhibition and STAT5 activation in vivo will offer a better look at the future feasibility of this therapeutic approach. Consideration of tumor type and prognostic outlook of STAT5 activation will have to be considered since STAT5 is oncogenic in some cases. STAT3 also has a range of biological functions, in addition to transcriptional activity, that will need to be considered when inhibiting STAT3. Since constitutive STAT3 is often associated with TNBC and other aggressive breast cancer subtypes that currently lack effective therapies, using STAT5 to modulate STAT3 activity could be an interesting avenue to pursue.

## Abbreviations

The following abbreviations are used in this manuscript:

ABCB1	ATP binding cassette subfamily B member 1
BCL6	B-cell lymphoma 6
CPT1B	Carnitine palmitoyltransferase-1B
EGF	Epidermal growth factor
EMT	Epithelial mesenchymal transition
ER	Estrogen receptor
ERRα	Estrogen-related receptor α
FGFR-2	Fibroblast growth factor receptor 2
GLI	Glioma associated oncogene homolog 1
HER2	Human epidermal growth factor receptor 2
HMGN2	High mobility group nucleosome binding domain 2
HMGN5	High mobility group nucleosome binding domain 5
IGF	Insulin-like growth factor
IL6	Interleukin 6
ISG20	Interferon stimulated exonuclease gene 20
JAK	Janus kinase
LIF	Leukemia inhibitory factor
LIFR	Leukemia inhibitory factor receptor
Naa10p	N-a-acetyltransferase 10 protein
NOX5-L	NADPH oxidase 5 long form
OCT1	Octamer transcription factor 1
OSM	Oncostatin M
OSMR	Oncostatin M receptor
PD-L1	Programmed death ligand 1
PIAS	Protein inhibitor of activated STATs
PR	Progesterone receptor
SOCS	Suppressor of cytokine signaling
STAT3	Signal transducer and activator of transcription
STAT5	Signal transducer and activator of transcription
TNBC	Triple negative breast cancer
TNFRSF1A	Tumor necrosis factor receptor superfamily member 1A
VGLL4	Vestigial like family member 4
YAP1	Yes-associated protein 1
